# Simultaneous Robot–World and Hand–Eye Calibration without a Calibration Object

**DOI:** 10.3390/s18113949

**Published:** 2018-11-15

**Authors:** Wei Li, Mingli Dong, Naiguang Lu, Xiaoping Lou, Peng Sun

**Affiliations:** 1 Institute of Information Photonics and Optical Communications, Beijing University of Posts and Telecommunications, Beijing 100876, China; liweikilary@bupt.edu.cn (W.L.); sunpeng@bistu.edu.cn (P.S.); 2 Key Laboratory of the Ministry of Education for Optoelectronic Measurement Technology and Instrument, Beijing Information Science and Technology University, Beijing 100192, China; dongml@bistu.edu.cn (M.D.); louxiaoping@bistu.edu.cn (X.L.)

**Keywords:** robot–world calibration, hand–eye calibration, calibration object, Kronecker product, sparse bundle adjustment

## Abstract

An extended robot–world and hand–eye calibration method is proposed in this paper to evaluate the transformation relationship between the camera and robot device. This approach could be performed for mobile or medical robotics applications, where precise, expensive, or unsterile calibration objects, or enough movement space, cannot be made available at the work site. Firstly, a mathematical model is established to formulate the robot-gripper-to-camera rigid transformation and robot-base-to-world rigid transformation using the Kronecker product. Subsequently, a sparse bundle adjustment is introduced for the optimization of robot–world and hand–eye calibration, as well as reconstruction results. Finally, a validation experiment including two kinds of real data sets is designed to demonstrate the effectiveness and accuracy of the proposed approach. The translation relative error of rigid transformation is less than 8/10,000 by a Denso robot in a movement range of 1.3 m × 1.3 m × 1.2 m. The distance measurement mean error after three-dimensional reconstruction is 0.13 mm.

## 1. Introduction

With the progress of robot-vision-system advanced technology, it is necessary to evaluate the geometric relationships among the robot, sensors, and a reference frame. This problem is usually called “robot–sensor calibration”, and it has been an active area of research for almost 40 years [1]. As research has progressed, the applications of robot–sensor calibration have extended into many domains, such as automobile assembly, robot navigation, and endoscopic surgery. As reported previously [2], the most widespread mathematical representations for the robot–sensor calibration problem can all be grouped into two categories: *AX* = *XB* and *AX* = *ZB*.

The first class, and the most common robot–sensor calibration problem, is hand–eye calibration *AX* = *XB*, which was proposed by Tsai et al. [3] and Shiu et al. [4]. The earliest solution strategy estimated the rotation and translation with respect to homogeneous transformation *X* separately [5,6]. However, it was found that such a method would produce rotation error spread in the process of the translation estimation. In later strategies, both the rotation and translation with respect to homogeneous transformation *X* are solved simultaneously [7,8,9]. The above calibration methods solve the hand–eye relationship with different parametric approaches, such as the quaternion, dual quaternion, and Kronecker product, which are all inseparable from a known calibration object. However, there are many situations in which using an accurately-manufactured calibration object is not convenient, or is not possible at all. Indeed, due to restrictions in limited onboard weight or strictly sterile conditions, it may be inadvisable to use a calibration object in applications such as mobile robotics or endoscopy surgery. Thus, later, an approach for getting rid of the calibration object based on the structure from motion (SFM) technique was proposed by Andreff et al. [10], and this method—also named “extended hand–eye calibration”—could handle a wider range of problems. Subsequently, a similar approach was presented in [11], where a scale factor was included into quaternion and dual quaternion formulation. Ruland et al. [12] proposed a branch-and-bound parameter space search method for this extended hand–eye calibration problem, which guaranteed the global optimum of rotational and translational components with respect to a cost function based on reprojection errors. In [13,14], Heller et al. firstly utilized second order cone programming (SOCP) to calculate the hand–eye relationship and scale factor based on the angular reprojection error, and then exploited a branch-and-bound approach to minimize an objective function based on the epipolar constraint. However, this branch-and-bound search process was very time intensive. Soon afterwards, Zhi et al. [15] proposed an improved iterative approach to expedite the calculation speed concerning the above extended hand–eye calibration problem. Recently, with some consideration for the asynchrony of different sensors with respect to sampling rates and processing time by an online system, Li et al. [16] presented a probabilistic approach to solve the correspondence problem of data pairs (*A_i_*, *B_i_*). However, this method has not been tested with a real robotic system. Due to the narrow range of motion allowed by the surgical instrument in minimally-invasive surgery, Pachtrachai et al. [17] replaced planar calibration object with the CAD models of surgical tools in the process of hand–eye calibration; thus, the instrument 3D pose tracking problem has to be addressed in advance.

The second class of robot–sensor calibration problems is the form *AX* = *ZB*, which was first derived by Zhuang et al. [18]. This equation allowed the simultaneous estimation of the transformations from the robot-base coordinates to the world frame *Z*, and from the robot-gripper coordinate to the camera coordinate *X*. There are also two ways to approach the robot–world and hand–eye calibration problem. In the first, the rotation and translation components associated with *X* and *Z* are calculated separately based on dual quaternion and Kronecker product [19,20]. In the second, the rotation and translation components are computed simultaneously based on the quaternion and Kronecker product [21,22]. Additionally, in order to obtain a globally-optimal solution, Heller et al. [23] utilized convex linear matrix inequality (LMI) relaxations to simultaneously solve robot–world and hand–eye relationships. Very recently, in [24,25], Tabb et al. proposed a bundle adjustment-based approach, which is similar to the bundle adjustment partition of our algorithm. However, the main difference is that Tabb’s approach works based on a chessboard target, which our approach does not need.

To the best of the authors’ knowledge, all approaches for robot–world and hand–eye calibration are implemented with an external calibration object. However, it is necessary to further research the solving of the robot–world and hand–eye calibration problem without a calibration object, which is named “extended robot–world and hand–eye calibration” in this paper. Our work on this problem is motivated by two particular situations. The first is the use of a robot-mounted camera for multi-view, high-quality reconstruction of general objects by a rescue or endoscopic robot, where the reconstruction outcomes depend on the feature-matching accuracy instead of a 2D chessboard target. The second situation is the use of the same robot-mounted camera for large-scale digital photogrammetry under industrial conditions, such as aircraft and shipbuilding assembly sites. In this situation, in view of the limit of single measurement range, the measurement surface is segmented into several small parts. A robot with two linear guides is used to define and record the placement of the optical measurement system in front of the measurement surface. The imaging system, based on retroreflective targets (RRTs), is mounted on the robot gripper as an end effector, and non-experts can be allowed to complete the calibration and acquire the three-dimensional (3D) coordinates of the target points attached to the measurement surface from a remote location.

For these particular situations, an extended robot–world and hand–eye calibration approach without a calibration target is proposed for a robotic visual measurement system. At first, our approach improves the *AX* = *ZB* mathematical model by supposing that different camera poses comprise up to an unknown scale factor, and propose a fast linear method to give an initial estimate to the calibration equation. Then, we combine space intersection and sparse bundle adjustment to refine the robot–world and hand–eye transformation relationship, as well as 3D reconstruction, simultaneously. Finally, we demonstrate the effectiveness, correctness, and reliability of our approach with relevant synthetic and real data experiments.

## 2. Problem Formulation

### 2.1. Initial Estimate

**Figure 1:**
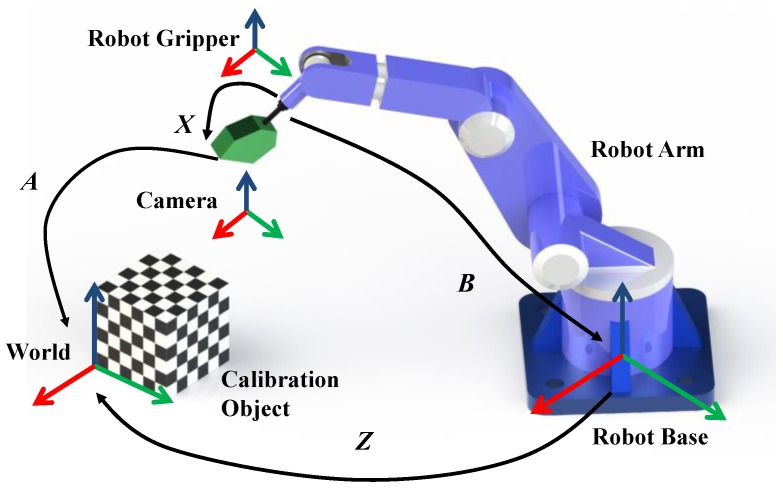
The robotic system of robot–world and hand–eye calibration.

Supposing that we have an arbitrary position of the robotic system, from Figure 1, we can define:

(1) AX=ZB

The homogeneous transformation matrix *A* is obtained by calibrating extrinsic camera parameters with respect to a fixed calibration object. The homogeneous transformation matrix *B* is computed using the internal-link forward kinematics of the robot arm. *X* is the robot-gripper-to-camera rigid transformation, which is always constant, as the camera is rigidly mounted on the robot gripper, and *Z* is the robot-base-to-world rigid transformation.

Now, let *R_A_*, *R_B_*, *R_X_* and *R_Z_* ∈ *SO*(3) denote the respective 3 × 3 rotational matrices of *A*, *B*, *X* and *Z*. Let *t_A_*, *t_B_*, *t_X_*, and *t_Z_* denote the respective 3 × 1 translational vectors, which are measured using the same scale unit. Equation (1) can be easily decomposed into a rotational matrix equation and translational vector equation:

(2) $$R_{A}R_{X}=R_{z}R_{B},R_{A}t_{X}+t_{A}=R_{z}t_{B}+t_{z}$$

If there is no 3D calibration object, such as in 2D-to-3D correspondences, we have to use SFM to estimate camera poses based on 2D-to-2D correspondences only. However, due to the lack of a given scale factor, SFM can reconstruct the structure of the scene and the camera poses up to an unknown scale factor. Of course, we can introduce an explicit scaling factor to the robot–world and hand–eye calibration equation, with reference to Andreff [10]. Equation (2) can be transformed into

(3) $$R_{A}R_{X}=R_{z}R_{B},R_{A}t_{X}+\alpha t_{A}=R_{z}t_{B}+t_{z}$$

The Equations (3) can be used to formulate an objective function *f*(·) for non-linear optimization, which is based on the objective function for standard robot–world and hand–eye calibration proposed by [21]:

(4) $$\begin{array}{ll} f\left(q_{x},q_{z},t_{X},t_{Z},\alpha \right) & =\lambda_{1}\sum_{i=1}^{N}{\left\|Q\left(q_{A_{i}}\right)q_{x}-W\left(q_{z}\right)q_{B_{i}}\right\|}^{2}+\lambda_{2}{\sum_{i=1}^{N}\left\|W{\left(q_{A_{i}}\right)}^{T}Q\left(q_{A_{i}}\right)t_{X}+\alpha t_{A_{i}}-W{\left(q_{z}\right)}^{T}Q\left(q_{z}\right)t_{B_{i}}-t_{Z}\right\|}^{2}\\ & +\lambda_{3}{\left(1-q_{x}{\bar{q}}_{x}\right)}^{2}+\lambda_{4}{\left(1-q_{z}{\bar{q}}_{z}\right)}^{2} \end{array}$$

where *W*(*q*)^T^*Q*(*q*) is an orthogonal matrix for quaternion *q*, and the parameters *λ*_1_ through *λ*_4_ are regularization factors (e.g., *λ*_1_ = *λ*_2_ = 1 and *λ*_3_ = *λ*_4_ = 10^6^). In addition to scale factor α, the rotations and translations associated with *X* and *Z* can be estimated simultaneously by solving Equation (4).

$$W{\left(q\right)}^{T}Q\left(q\right)=\left[\begin{array}{cccc} 1 & 0 & 0 & 0\\ 0 & q_{0}^{2}+q_{1}^{2}-q_{2}^{2}-q_{3}^{2} & 2\left(q_{1}q_{2}-q_{0}q_{3}\right) & 2\left(q_{1}q_{3}+q_{0}q_{2}\right)\\ 0 & 2\left(q_{1}q_{2}+q_{0}q_{3}\right) & q_{0}^{2}-q_{1}^{2}+q_{2}^{2}-q_{3}^{2} & 2\left(q_{2}q_{3}-q_{0}q_{1}\right)\\ 0 & 2\left(q_{1}q_{3}-q_{0}q_{2}\right) & 2\left(q_{2}q_{3}+q_{0}q_{1}\right) & q_{0}^{2}-q_{1}^{2}-q_{2}^{2}+q_{3}^{2} \end{array}\right]$$

Referring to [20], we can also obtain the separable solutions to the robot–world and hand–eye calibration problem by Kronecker product. Since *R_A_* and *R_B_* are both an orthogonal matrix, the orientation component of Equation (3) can also be represented as:

(5) $$\left(\begin{array}{cc} nI & -\sum_{j=1}^{n}R_{B_{i}}⊗R_{A_{i}}\\ -\sum_{j=1}^{n}R_{B_{i}}^{T}⊗R_{A_{i}}^{T} & nI \end{array}\right)\left(\begin{array}{c} vec\left(R_{Z}\right)\\ vec\left(R_{X}\right) \end{array}\right)=\left(\begin{array}{c} 0\\ 0 \end{array}\right)$$

Those vectors of Equation (5) can efficiently be computed by singular value decomposition (SVD). The symbol ⊗ denotes the Kronecker product, and the column vector operator *vec* reorders the coefficients of a (*m* × *n*) matrix *A* into an *mn* vector *vec*(*A*) = (*a*_11_, …, *a*_1_*_n_*, *a*_21_, …, *a_mn_*) [26]. Once *R_Z_* is calculated by Equation (5), *t_X_*, *t_Z_* is the solution to the linear system:

(6) $$\left[\begin{array}{ccc} R_{A} & -I_{3\times 3} & t_{A} \end{array}\right]\left[\begin{array}{c} t_{X}\\ t_{Z}\\ \alpha \end{array}\right]=R_{Z}t_{B}$$

The solution to *t_X_*, *t_Z_* and α can be easily determined by least square technique. However, the variety of the additional scale factor α will bring instability into Equation (3). To overcome this problem, we propose a novel solution through eliminating α based on the Kronecker product. We define t_A_* as a skew-symmetric matrix corresponding to *t_A_*, which can be denoted as

$$t_{A}{}^{*}=\left[\begin{array}{ccc} 0 & -t_{3} & t_{2}\\ t_{3} & 0 & -t_{1}\\ -t_{2} & t_{1} & 0 \end{array}\right]$$

Since the scale factor α has no influence on the computation of rotation, the rotational part of Equation (3) is the same, and the translational part of Equation (3) is multiplied on both sides by the skew-symmetric t_A_*. Obviously, t_A_* *t_A_* = [0, 0, 0]^T^, and the new equation can be formulated as follows:

(7) $$R_{A}R_{X}R_{B}{}^{T}=R_{z}\;,\;\;t_{A}{}^{*}R_{A}t_{X}=t_{A}{}^{*}R_{z}t_{B}+t_{A}{}^{*}t_{z}$$

By using the Kronecker product theory, and if *AXB* = *C* for an unknown matrix *X*, then the equation can be rewritten as a linear system:

$$vec\left(AXB\right)=\left(B^{T}⊗A\right)vec\left(X\right)=vec\left(C\right)$$

Thus, Equation (7) can be reconstituted into

(8) $$\left[\begin{array}{cccc} R_{B}⊗R_{A} & -I_{9} & 0_{9\times 3} & 0_{9\times 3}\\ 0_{3\times 9} & t_{B}{}^{T}⊗t_{A}{}^{*} & -t_{A}{}^{*}R_{A} & t_{A}{}^{*} \end{array}\right]\left[\begin{array}{c} vec\left(R_{X}\right)\\ vec\left(R_{Z}\right)\\ t_{X}\\ t_{Z} \end{array}\right]=\left[\begin{array}{c} 0_{18\times 1}\\ 0_{6\times 1} \end{array}\right]$$

Obviously, the solution of the linear system (8) can be solved by SVD, and since *R_X_* and *R_Z_* are rotational matrices, there is a proportionality constraint in that the *R_X_* and *R_Z_* have a determinant value of 1. Thus, the unique solution can be determined. Supposing that the solution of the linear system (8) is proportional to the right singular vector v corresponding to the minimum singular value, the resulting *R_X_* and *R_Z_* can be estimated as

(9) $$R_{X}=\omega V_{X},R_{Z}=\varphi V_{Z}$$

where *V_X_* = *vec*^-1^(v_1:9_), *V_Z_* = *vec*^−1^(v_10:18_), *vec*^−1^ is defined as the inverse operator to *vec*, and the proportionality constants are

$$\omega =\mathrm{sign}\left(V_{X}\right)\det{\left(V_{X}\right)}^{-\frac{1}{3}},\varphi =\mathrm{sign}\left(V_{Z}\right)\det{\left(V_{Z}\right)}^{-\frac{1}{3}}$$

Therefore, the calculated robot–world and hand–eye translation vectors are

(10) $$t_{X}=\omega vec\left(v_{19:21}\right),t_{Z}=\varphi vec\left(v_{22:24}\right)$$

However, the calculated matrices *R_X_* and *R_Z_* may be not strictly orthogonal due to noise. Therefore, to ensure that they are indeed rotations, it is necessary to re-orthogonalize the computed rotation matrices.

### 2.2. Data Selection

SFM is a general method for obtaining camera poses from image correspondences, and mainly consists of feature point detection, feature point matching, camera pose calibration, and reconstruction. Given two view feature points that are coarse matching, there are a significant number of outliers in the estimated transformations of camera poses, and these outliers will inevitably affect the accuracy of the initial estimate for extended robot–world and hand–eye calibration. RANSAC [27] is a simple but robust algorithm for outlier removal, which has been used widely in computer vision. In this section, we utilize it to enhance robustness of the initial estimate. Referring to the RANSAC method [15], we randomly select a certain number of two view image correspondences and solve the extended robot–world and hand–eye calibration equation by the linear system (8). Firstly, as three pairs of camera pose solutions are just enough to determine the unique robot–world and hand–eye transformation [20], three pairs of camera orientation results are treated as the minimum number required for this sample. Then, we predict ${\hat{A}}_{i}$ using Equation (2):

(11) $$R_{{\hat{A}}_{i}}=R_{z}R_{B_{i}}R_{X}{}^{T},\;\;\;\;\;\;t_{{\hat{A}}_{i}}=R_{z}t_{B_{i}}+t_{z}-R_{{\hat{A}}_{i}}t_{X}$$

So, the rotation error *e_R_* can be defined as follows:

(12) $$e_{R}={\left\|R_{A_{i}}-R_{{\hat{A}}_{i}}\right\|}_{2}$$

Because the predicted translation $t_{{\hat{A}}_{i}}$ and original translation $t_{A_{i}}$ may not be calculated based on the same scale factor, the translation error *e_t_* is defined as follows:

(13) $$e_{t}=\left|{\left\|t_{{\hat{A}}_{i}}\right\|}_{2}-\frac{\langle t_{A_{i}},t_{{\hat{A}}_{i}}\rangle }{{\left\|t_{A_{i}}\right\|}_{2}}\right|$$

In addition, we combine the rotation and translation errors as the total error. Considering that the translation unit is always set to millimeters, in order to balance the rotation and translation errors, we scale the translation error by 0.01, so the total error *e* is

(14) $$e=e_{R}+0.01*e_{t}$$

Finally, we calculate the total error *e* for all valid random samples, and determine the largest set of consistent pairs. In this section, we let the error threshold *e* be 0.01, and the maximum outlier ratio be 50%. It should be considered that this selection process is just an initial estimate. There is no need to spend substantial amounts of time for minor accuracy improvement, so we stop RANSAC when the maximum iteration limit reaches 100.

### 2.3. Sparse Bundle Adjustment

Following the initial estimation for robot–world and hand–eye transformation by the Kronecker product, which is solved by Equations (9) and (10), we employ bundle adjustment to jointly refine the robot–world, hand–eye transformations, and the reconstruction results simultaneously. Bundle adjustment is almost invariably solved as the last step of feature-based 3D reconstruction algorithms and motion estimation computer vision algorithms to obtain optimal solutions. Generally speaking, the goal in bundle adjustment is to minimize the overall reprojection error between the observed and predicted image points. The mathematical expression can be depicted as below: assume that *m* 3D points are seen in *n* views, and let *x_ij_* indicate the projection of the *i*th point on the *j*th image. Assume also that λ*_ij_* is equal to 1 if the *i*th point can be observed on the *j*th image, otherwise it is equal to 0. Moreover, assume that *A_j_* is the rigid homogeneous transformation from the *j*th image frame to the world frame and that *G_i_* is the predicted 3D *i*th point by space intersection, and let *P_j_*(·) be the predicted projection matrix of the *j*th image, including camera-intrinsic parameters. The bundle adjustment model minimizes the reprojection error with respect to all 3D points and camera parameters, specifically:

(15) $$\underset{P_{j},A_{j},G_{i}}{\min}\sum_{i=1}^{m}\sum_{j=1}^{n}\lambda_{ij}{\left\|x_{ij}-P_{j}\left(A_{j}^{-1}G_{i}\right)\right\|}_{2}$$

Problems that are substantially similar to problem (15) can typically be tackled with non-linear least-squares optimization routines such as the Levenberg–Marquardt or Gauss–Newton approaches. Conventional bundle adjustment methods solve the normal equations repeatedly with complexity *O*(*n*^3^) in the number of unknown parameters for each iteration. However, substantial time-saving can be achieved by taking advantage of the sparse block structure contained in the normal equation [28]. In this way, a software implementation of sparse bundle adjustment is proposed by Lourakis and Argyros [29].

In our experiment, we utilize their implementation to solve the extended robot–world and hand–eye calibration problem. In order to refine the initial guess of *X* and *Z* using sparse bundle adjustment, the homogeneous transformation *A_j_*(α) up to an unknown scale factor is substituted by the inverse Equation (1) *A_j_* = *ZB_j_X*^−1^, because the robot arm pose *B_j_*, which is calibrated before delivery, can provide the real metric units. Then, the point 3D initial coordinates can be calculated by space intersection or triangulation. Finally, the sparse bundle adjustment method optimizes the robot–world transformation *Z*, hand–eye transformation *X*, and target point 3D coordinates *G_i_* simultaneously, while keeping the robot motions *B_j_* and camera-intrinsic parameters constant. Specifically, the sparse bundle adjustment model can be rewritten as:

(16) $$\underset{X,Z,G_{i}}{\min}\sum_{i=1}^{m}\sum_{j=1}^{n}\lambda_{ij}{\left\|x_{ij}-P_{j}\left(XB_{j}^{-1}Z_{j}^{-1}G_{i}\right)\right\|}_{2}$$

Note that the robot–world *Z* and hand–eye *X* transformations consist of 6 rotation parameters and 6 translation parameters, while each point consists of 3 position parameters. The total number of minimization parameters in Equation (16) equals 3*m* + 12. According to specific needs, we can set a termination condition for iteration. The iterations are terminated when the estimated robot–world translation changes by less than 10^−3^ mm, or the reconstruction 3D points changes by less than 10^−3^ mm, compared to that of the last iteration, or reaches the maximum limit of iterations, which is ten in this paper.

## 3. Experiments

This section validates the proposed method for the extended robot–world and hand–eye calibration problem both on synthetic and real datasets. For the data comparison, with some considerations, one could not expect that the method without a calibration object would obtain results as accurate as the method with a calibration object. In this paper, our main purpose is that the estimation of the robot–world and hand–eye transformation is feasible without a calibration object. We refer to the means of data comparison of previous extended hand–eye calibration methods, such as those presented by Nicolas Andreff [10], Jochen Schmidt [11], and Jan Heller [13]. We present an experimental evaluation of the extended robot–world and hand–eye methods, in which the estimation of rotation, translation, and scale factor can be formulated using the Kronecker product [20], or quaternions [21], or reprojection error [25], and a standard robot–world and hand–eye calibration method [25] with chessboard pattern calibration was used as an approximate truth-value, since no ground truth is available to compare accuracy between different methods. For convenience, in the following experiments, the labels “Dornaika” and “Shah” stand for the estimation of rotation, translation, and scale factor using the quaternions Equation (4) or Kronecker product Equation (5), respectively. The label “KPherwc” stands for the proposed initial calibration method based on the Kronecker product Equation (8), and the label “BAherwc” stands for the proposed optimization approach based on sparse bundle adjustment Equation (16). VisualSFM [30]—a state-of-the-art, open-source SFM implementation—was used to obtain the camera poses for a general object in real-data experiments. All method results were obtained using a hybrid MATLAB 9.0 and C++ reference implementation, and we conducted the methods on an Intel Core i7-8750H processor running Linux.

### 3.1. Experiments with Synthetic Data

**Figure 2:**
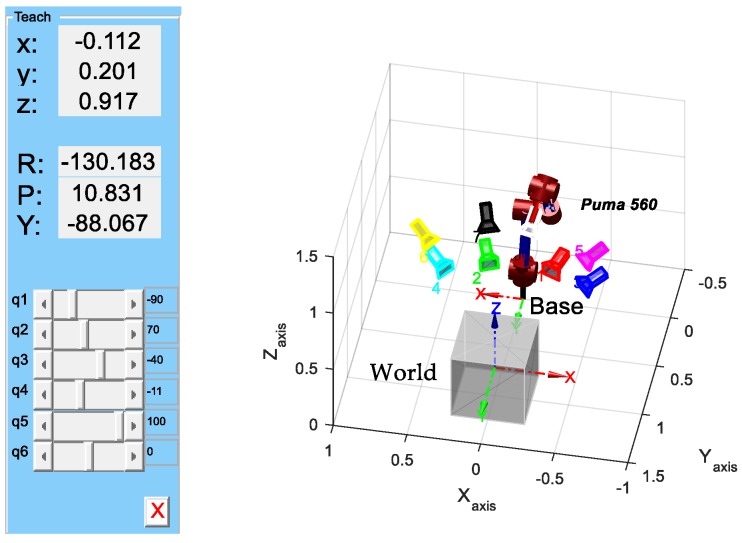
Schematic diagram of the synthetic experiment using the PUMA560 model.

In order to simulate the actual process of robot motions, considering that PUMA560 is the most classic robot arm kinematics model, and that this robot has been well studied and its parameters are very well known—it has been described as the “white rat” of robotics research [31]—referring to Zhuang [18], we used PUMA560 robot kinematics modeling and a camera imaging model to build a synthetic scene and a virtual camera. As shown in Figure 2, a red PUMA560 robot arm was constantly in movement with a different-colored camera attached to the end gripper. A synthetic scene consisting of 50 3D points was generated randomly into a gray cube with side length 0.5 m, and 8 different virtual camera poses set such that the cameras were faced approximately to the center of the cube were created. The intrinsic parameters of the virtual camera and Denavit–Hartenberg (DH) parameters of the PUMA 560 robot are separately listed in Table 1 and Table 2.

To test the performance of different methods against projection noise, the simulated data were conducted with the synthetic scene and a virtual camera. The scene 3D points were projected into the image plane after each position movement, but the projection points would be neglected if they were outside the image plane. In order to qualitatively analyze and evaluate the results of the synthetic experiment, we defined the error evaluation criteria associated with rotation and translation as follows:

$$e_{R}={\left\|\tilde{R}-R\right\|}_{2}\;\;\;\;\;\;e_{t}=\frac{{\left\|\tilde{t}-t\right\|}_{2}}{{\left\|\tilde{t}\right\|}_{2}}$$

where $\tilde{R}$ represents the true rotation, R represents the estimated rotation, $\tilde{t}$ represents the true translation, and *t* represents the estimated translation. In the synthetic experiment, since the nominal value for the robot–world and hand–eye transformation can be set up in advance, there is no need to use a standard robot–world and hand–eye calibration method [25] as an approximate truth-value. We set ${\left\|{\tilde{t}}_{X}\right\|}_{2}$ = 0.1 m and ${\left\|{\tilde{t}}_{Z}\right\|}_{2}$ = 1 m. The entire experiment is a four-step process. Firstly, considering that real-world feature point extraction is generally expected to have accuracy within 1 pixel, projection points in the synthetic experiment were corrupted by 6 different levels of Gaussian noise in the image domain with a standard deviation *η* ∈ [0, 1] pixel and a step of 0.2 pixel. Secondly, according to the synthetic scene, we defined the nominal value for the hand–eye transformation $\tilde{X}$ and the robot-to-world transformation $\tilde{Z}$ with constant translation ${\tilde{t}}_{X}$ and ${\tilde{t}}_{Z}$. Thirdly, we calculated a sequence of camera positions based on space resection, and the corresponding robot motions were calculated by *B* = *Z^−^*^1^*AX*. Considering that the noise of robot motion is determined after production, we added a constant noise (σ = 0.025 mm) to robot joint offset. Finally, we performed the homogeneous transformations *X* and *Z* with the above four different methods and compared their rotation and translation errors in the presence of various noise levels. For each noise level, 50 repeated experiments were done with randomly generated sets of data, and the final value was the mean of all 50 errors.

**Figure 3:**
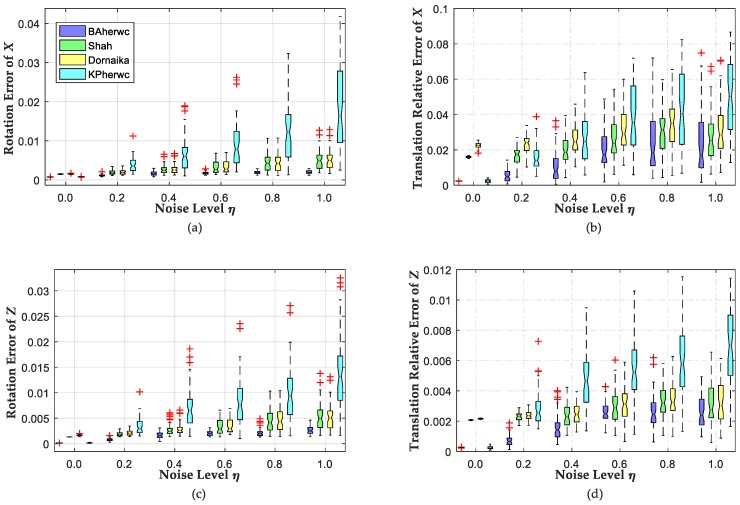
Error of estimated rotation and translation against different noise levels *η*: (**a**,**b**) The rotation and translation errors with regard to hand–eye transformation *X*; (**c**,**d**) The rotation and translation errors with regard to robot–world transformation *Z*.

Figure 3 illustrates the rotation and translation errors for each noise level using the boxplot. Clearly, our optimization method (“BAherwc”) exhibits the best behavior both in rotation and translation estimation of the transformation *X* and *Z*, whereas the proposed initial calibration method (“KPherwc”) performs worst under noise conditions; thus, it is extremely effective to refine the initial calibration results by follow-up sparse bundle adjustment. Meanwhile, the translation relative errors estimated by “Shah” are slightly better than those estimated by “Dornaika”. This is a result of the “Dornaika” method calculating the rotation and translation transformations regarding *X* and *Z* all in the same step. Due to noise, the estimated rotations may not be accurate representations of the rotation matrices, and thus, a set of nonlinear constraints have to be made for the rotation matrices; meanwhile, the estimated translations are not updated with the orthogonal restriction, which causes the larger positional errors that are illustrated in Figure 3.

### 3.2. Experiments with Real Datasets

In this experiment, a Denso VS-6577GM serial 6-DOF robot arm with a Nikon D300s digital SLR camera and an AF NIKKOR 20 mm lens was used to acquire the real data. Since no ground truth gripper–camera transformation is available in the real data, it is difficult to give direct error results about the computed robot–world and hand–eye transformation, such as for the synthetic data. Therefore, it is desirable to measure the quality of the calibration results between the camera and robot device in some indirect way. In the rest of this section, we arranged two different scenes to complete the accuracy assessment: scene A, with a general object, was used to show the general applicability of the proposed method compared to the standard robot–world and hand–eye calibration approaches, and scene B, a photogrammetric retro-reflective target was used to improve the feature point-locating accuracy and decrease the false match rate for calibrating the extrinsic camera. Before the experiment, we used [32] to calibrate the camera together with seven parameters of lens, so the images were undistorted prior to being further used in order to improve sparse bundle adjustment results.

#### 3.2.1. Dataset A

**Figure 4:**
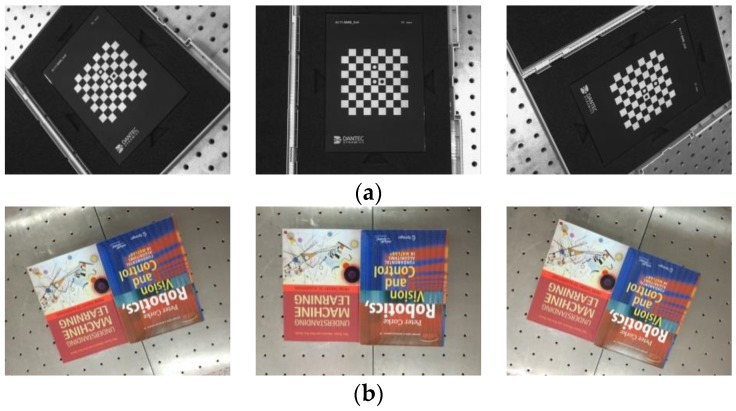
Sample images of calibration scenarios taken by the camera mounted on the gripper of the robot: (**a**) Chessboard pattern scene; (**b**) Books scene.

With dataset A, our main purpose is not to prove how high the accuracy of our method is, but to demonstrate the feasibility of estimating the robot–world and hand–eye transformation without a calibration object in a general scene. Two image sets were required for the performance of the different methods in real-world conditions, as shown in Figure 4. Some consideration for the absence of a ground truth is available in the real-data experiment. We cannot give errors between the real robot–world transformation and the computed one, just like in the synthetic experiment. Since the method with a calibration object can usually obtain more accurate results than the method without a calibration object, a chessboard pattern was firstly used for solving robot–world *Y*_bar_ and hand–eye *X*_bar_ transformation simultaneously by the Tabb method [25], which could be assumed to give an approximate true value for the present. Afterwards, we removed the chessboard pattern, and used books as the object instead. We used the above “Dornaika”, “Shah”, “KPherwc”, and “BAherwc” methods to calculate the homogeneous transformation *X*_scene_ and *Y*_scene_ with the general object of books. Finally, we compared their results to the approximate true value *X*_bar_ and *Y*_bar._ The errors of robot–world and hand–eye relationships are defined as follows:

$$E_{X}={\left\|X_{\mathrm{bar}}-X_{\mathrm{scene}}\right\|}_{2}\;\;\;\;\;\;E_{Y}={\left\|Y_{\mathrm{bar}}-Y_{\mathrm{scene}}\right\|}_{2}$$

**Figure 5:**
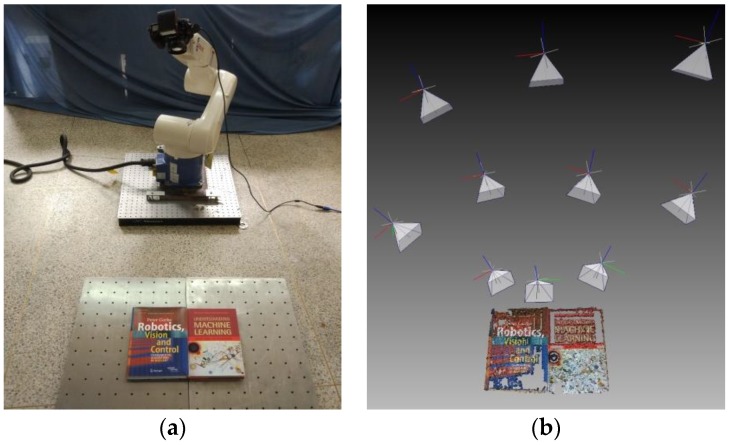
General object data set experiment: (**a**) Denso robot arm with Nikon camera; (**b**) 3D model output after bundle adjustment.

Figure 5a shows that the robot gripper carrying the camera took a series of photos around the center of the books. The positions of the gripper were adjusted with ten different locations, and it was ensured that the entirety of the books were in the view in every frame. The camera was set to manual mode, and images of 4288 × 2848 pixels were taken using a PC remote control. After all the photos were taken, a fast open-source SFM implementation was used to obtain the camera pose *A_i_*(*α*), and the robot motion transformation *B_i_* was obtained from the known gripper-to-robot-base transformations. Then we computed robot–world and hand–eye transformation using the above four methods. Figure 5b shows the resulting 3D model output from bundle adjustment, containing 49,352 points in space, and the poses of all the ten cameras. Due to a high number of correspondences, only every hundredth member of the set of corresponding image points was used in our experiment.

Table 3 summarize the results obtained with the two image sets mentioned above. Compared with other similar methods, it can be seen that our “BAherwc” method is nearest to the results of Tabb method [25] based on the chessboard pattern calibration. This is because in the “BAherwc” method, it was initialized by the results from the “KPherwc” method; then, the reprojection error is directly minimized, like in Tabb reprojection [25]. On the other hand, in the “Dornaika” and “Shah” method, the variety of the scale factor will bring instability into the solution of the robot–world and hand–eye transformation during the SFM implementation. Of course, one could not expect to obtain results as accurate as with Tabb’s standard calibration. However, depending on the different application, the advantages of the proposed extended method may outweigh this drawback. It is especially true for mobile robotics or endoscopy setups that we have in mind, where robot–world and hand–eye calibration has to be performed under specific situations, due to the restrictions in limited onboard weight or the strict sanitary conditions. In order to achieve a rough qualitative analysis, we also measured the translation from the gripper to the camera lens center by hand with the known mechanical structure of the gripper and join parts, which is approximated to [0, 58, 66] mm. The estimated translation by our “BAherwc” approach is [0.183, 57.326, 64.910] mm, which is close to the result of the previous physical measurement, showing the validity of the obtained results.

#### 3.2.2. Dataset B

**Figure 6:**
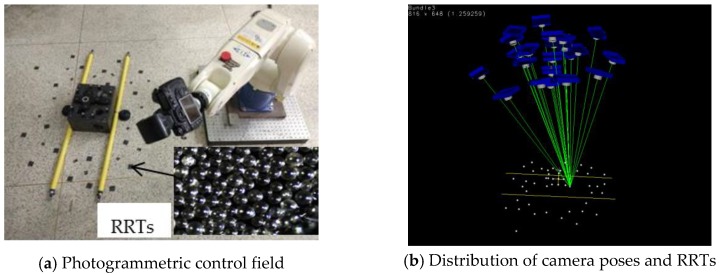
Photogrammetric scene data set experiment: (**a**) Photogrammetric control field; (**b**) Distribution of camera pose and target points.

In dataset B, our main purpose is to provide a mobile benchmark for large-scale digital photogrammetry under industrial conditions, which needs a robot to move along the guide rail to complete multi-station stitching measures. In order to reduce noise disturbance caused by SFM, we used photogrammetric retro-reflective targets (RRTs) to obtain accurate feature point matching. RRTs consist of a dense arrangement of small glass beads (Figure 6a bottom-right), as the name would suggest, which have good retro-reflective performance. The reflected light intensity in the light source direction is up to hundreds of times larger than the general diffuse reflection target. Thus, it is easy to obtain a subpixel level locating accuracy of feature points in the complex background image. As indicated in Figure 6a, dozens of RRTs and two yellow invar alloy scale bars S_1_ and S_2_ constructed a photogrammetric control field, and two 6 mm diameter coded RRTs were rigidly fixed on the yellow scale bar end, for which the distance had been accurately measured by a laser interferometer. Furthermore, coded RRTs can be encoded using specific pattern of distribution, which can actualize the automatic image matching of corresponding points. Then, the robot gripper carrying the camera took a series of photos around the center of the photogrammetric field, ensuring that the entire RRTs were in the camera view in every frame. Afterwards, we used the Hartley 5-point minimal relative pose method [33] and photogrammetry bundle adjustment to calibrate and optimize the extrinsic camera parameters.

Figure 6b shows the distribution of camera pose and RRTs. After bundle adjustment, the cameras were moved to 20 different poses faced to the RRTs and scale bars, and a Denso robot was moved in volume of 1.3 m × 1.3 m × 1.2 m. In view of photogrammetric relative orientation yields a high precision camera poses *A_i_*(*i* = 1, …, 20), we solved hand–eye transformation *X* and the robot-to-world transformation *Z* by means of three methods (the “Dornaika”, “Shah”, and our “BAherwc” method) based on existing camera pose *A_i_* and robot motion *B_i_*. Then, the predictive camera poses ${\hat{A}}_{i}$ can be inverse-computed with Equation (1):

$${\hat{A}}_{i}=ZBX^{-1}$$

In this section, the discrepancy between ${\hat{A}}_{i}$ and *A_i_* is supposed to an accuracy assessment basis of robot–world and hand–eye calibration. Considering the difference of scale factor between ${\hat{t}}_{A_{i}}$ and $t_{A_{i}}$, all translations are normalized beforehand, and the mean errors of all motions (from 1 to 20) are computed in rotation and translation. The rotation and translation relative errors are described as:

$$e_{R}={\left\|R_{A_{i}}-{\hat{R}}_{A_{i}}\right\|}_{2}\;\;\;\;e_{t}={\left\|\frac{t_{A_{i}}}{{\left\|t_{A_{i}}\right\|}_{2}}-\frac{{\hat{t}}_{A_{i}}}{{\left\|{\hat{t}}_{A_{i}}\right\|}_{2}}\right\|}_{2}$$

Comparisons of the accuracy in rotation and translation for photogrammetric scene data set are provided in Table 4. It can be seen that our method “BAherwc” is almost to half an order of magnitude better than the other methods with regard to both in rotation and translation estimation. The rotation error is less than 5/10,000, and translation relative error is less than 8/10,000. Our optimization method “BAherwc” outperforms the “Dornaika” and “Shah” methods, mainly because the feature extraction and matching accuracy of retroreflective targets is significantly higher than that of the general targets used by SFM; this is an expected behavior, as the “BAherwc” method, by minimizing overall reprojection error, depends on the feature extraction accuracy. This experiment might have confirmed the validity of our approach for the calibration of transformation parameters between the camera and the robot device based on the digital photogrammetric system.

Since the two invar alloy scale bars, S_1_ and S_2_, provided both feature correspondences and a distance measurement reference, we evaluated our “BAherwc” approach in the relative accuracy of 3D reconstruction by using distance measurement. The average distance measurement errors of the two scale bars are given in the following Table 5. For comparison, ${\hat{S}}_{1}$ and ${\hat{S}}_{2}$ are defined as calculated values based on our “BAherwc” method as reconstruction result byproduct. The distance measurement errors are described as:

$$e_{s}=\left|S_{i}-{\hat{S}}_{i}\right|,i=1,2$$

**Figure 7:**
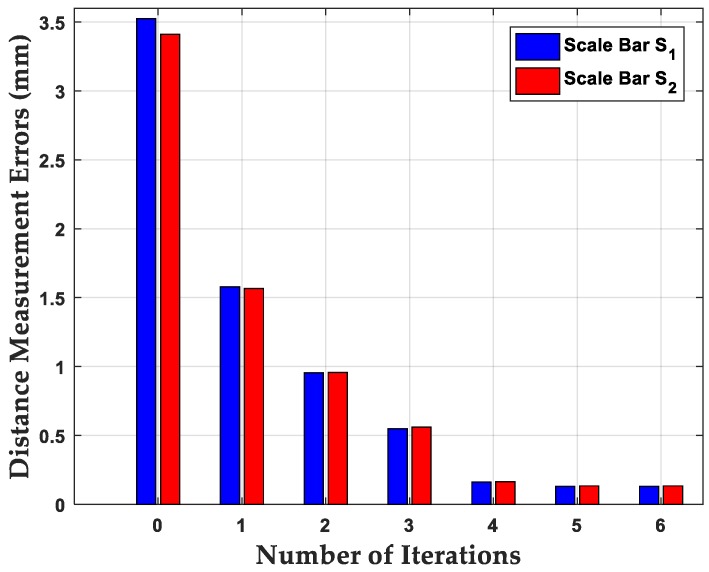
Distance estimation error iteration at each iteration by bundle adjustment.

Finally, to show the iterative process of our bundle adjustment method, Figure 7 illustrates the distance estimation error variances of the scale bars S_1_ and S_2_ at each iteration. One can see that although the initial reconstruction results are clearly inaccurate, the reconstruction errors after finite iteration still converge, and the final differences between the nominal value and measurement value of the two scale bars are close to 0.1 mm. Given that offline photogrammetry systems offer the highest precision and accuracy levels, the precision of feature point measurement can be as high as 1/50 of a pixel, yielding typical measurement precision on the object in the range of 1:100,000 to 1:200,000 [34], with the former corresponding to 0.01 mm for an object of 1 m in size. The absolute accuracy of length measurements is generally 2–3 times less (e.g., about 0.025 mm for a 1-m long object) than the precision of object point coordinates, which expresses the relative accuracy of 3D shape reconstruction. The relative accuracy of reconstruction by our bundle adjustment method is also influenced by the robot arm, and it can be improved by follow-up photogrammetric network design.

## 4. Conclusions

In this paper, we present an extended approach for robot–world and hand–eye calibration without the need for a calibration object. In order to obtain the calibration data, we use two kinds of extrinsic calibrations: one for computer vision system with SFM, and the other for the digital photogrammetric system with RRTs. These two calibration methods can both estimate the extrinsic camera parameters lacking a known scales factor. Meanwhile, the robot end gripper pose is computed using the manipulator’s forward kinematics, whose parameters are generally supposed to be known. Then, we use a fast initial estimation for extended robot–world and hand–eye calibration based on the Kronecker product. After the initial guess, to further improve the calibration results, we used sparse bundle adjustment to optimize the robot–world and hand–eye transformation relationship along with reconstruction. Finally, to evaluate and verify the feasibility of the proposed method, four accuracy assessment solutions were designed in the synthetic-data and real-data experiments. It is shown that our “BAherwc” approach can maintain a certain accuracy and robustness without a calibration object under the lower noise disturbance, and the Denso VS-6577GM, rigidly mounted to the floor, can obtain relatively reliable reconstruction results for follow-up photogrammetry stitching measures. In the future, we will move the industrial robot along the guide rail to expand the measurement range of the calibration procedures.

## Figures and Tables

**Table 1:** Intrinsic parameters of the virtual camera for the synthetic experiment.

Intrinsic Parameter	Image Resolution	Focal Length	Principle Point Offsets	Affine Distortion	Radial Distortion and Decentering Distortion
Value	4288 × 2848 pixels	20 mm	(0.1, 0.1) mm	0	0

**Table 2:** Denavit–Hartenberg parameters of the PUMA560 robot for the synthetic experiment.

Joint	q*_i_*/(°)	d*_i_*/m	a*_i_*/m	α*_i_*/(°)	Offset/(°)
1	q_1_	0	0	0	σ_1_
2	q_2_	0.2435	0	−90	σ_2_
3	q_3_	−0.0934	0.4318	0	σ_3_
4	q_4_	0.4331	−0.0203	90	σ_4_
5	q_5_	0	0	−90	σ_5_
6	q_6_	0	0	90	σ_6_

**Table 3:** Error comparison for the general object data set without a chessboard pattern as benchmark (Unit: mm).

Approach	Dornaika	Shah	KPherwc	BAherwc
Hand–eye transformation error *E_X_*	3.945	2.337	3.409	1.145
robot–world transformation error *E_Y_*	6.001	3.751	4.544	1.808

**Table 4:** Error comparison in rotation and translation for the photogrammetric scene dataset.

Approach	Rotation Error *e_R_*	Translation Error *e_t_*
Dornaika	0.0023	0.0033
Shah	0.0015	0.0017
BAherwc	0.00047	0.00076

**Table 5:** The average distance measurement errors of scale bars (Unit: mm)

Scale Bar	Nominal Value	Measurement Value	Distance Measurement Error *e_s_*
S_1_	1096.037	1095.906	0.131
S_2_	1096.057	1095.923	0.134
